# Scalable and CMOS compatible silicon photonic physical unclonable functions for supply chain assurance

**DOI:** 10.1038/s41598-022-19796-z

**Published:** 2022-09-19

**Authors:** Farhan Bin Tarik, Azadeh Famili, Yingjie Lao, Judson D. Ryckman

**Affiliations:** Holcombe Department of Electrical and Computer Engineering, Clemson University, Clemson, SC 29634 USA

**Keywords:** Integrated optics, Silicon photonics, Photonic devices

## Abstract

We demonstrate the uniqueness, unclonability and secure authentication of *N* = 56 physical unclonable functions (PUFs) realized from silicon photonic moiré quasicrystal interferometers. Compared to prior photonic-PUF demonstrations typically limited in scale to only a handful of unique devices and on the order of 10 false authentication attempts, this work examines > 10^3^ inter-device comparisons and false authentication attempts. Device fabrication is divided across two separate fabrication facilities, allowing for cross-fab analysis and emulation of a malicious foundry with exact knowledge of the PUF photonic circuit design and process. Our analysis also compares cross-correlation based authentication to the traditional Hamming distance method and experimentally demonstrates an authentication error rate AER = 0%, false authentication rate FAR = 0%, and an estimated probability of cloning below 10^−30^. This work validates the potential scalability of integrated photonic-PUFs which can attractively leverage mature wafer-scale manufacturing and automated contact-free optical probing. Such structures show promise for authenticating hardware in the untrusted supply chain or augmenting conventional electronic-PUFs to enhance system security.

## Introduction

Physical unclonable functions (PUFs) have garnered significant attention within the micro-electronics and hardware security communities due to their ability to provide chip-unique fingerprints or secret keys which provide a foundation for performing many cryptographic applications^1–3^. As electronic-PUFs have continued to mature^4^, their prospective limitations and vulnerabilities have become increasingly important factors to address and/or circumnavigate. Along this vein, photonic-PUFs have witnessed renewed and increasing interests in recent years as they attractively offer an inherently non-electronic platform with rich underlying physics and large information capacity. Photonic-PUFs are also uniquely compatible with non-contact optical probing and have been utilized within optical communication links^5,6^, allowing them to provide increasingly distinct functionalities. These and future iterations of photonic-PUFs are expected to facilitate cryptographic applications such as secure authentication, identification, and communication through a variety of prospective device formats, i.e., passive/active, all-optical, electronic-photonic, and quantum-optical.

To date a variety of “non-integrated" photonic PUFs have been demonstrated based on laser speckle^7^, irregular surface textures^8^, single and multimode optical fibers^9,10^, plasmonic nanoparticles^11^ and organic light emitters^12^. However, such approaches lack integration and nearly all require precisely controlled optical alignment, tilt, polarization, temperature, and/or stable 2D spatially resolved optical imaging to measure and authenticate. In addition to increasing ‘intra-chip’ variations owing to enhanced environmental or probing sensitivities, non-integrated solutions often lack scalability and are hindered by their “inconvenient compatibility with current complementary metal–oxide–semiconductor (CMOS) fabrication processes”^2^.

On the other hand, following the recent maturation and successful commercialization of integrated silicon photonics^13^, CMOS compatible integrated photonic-PUFs have begun to emerge as a promising and potentially scalable PUF platform^5,14–16^. Such integrated PUFs could be employed in modern silicon photonic process flows or could ultimately be co-integrated with microelectronics through ‘zero-change CMOS’ design principles^17^. Recent examples of integrated photonic-PUFs include chaotic nonlinear microresonators^5,14^, Mach–Zehnder interferometer (MZI) networks^16,18^, and our demonstration of quasicrystal interferometer (QCI) circuits complete with on-chip polarization and mode filters^15^. However, most photonic-PUFs reported to date have been realized and studied in very limited quantities, i.e., ranging from only a few devices^14,15^ up to roughly one dozen^19^. As photonic-PUFs push from proof-of-concept devices toward practical and scalable security solutions, it is increasingly important to examine and validate their unclonable characteristics on larger scales.

In our prior work, we have demonstrated a proof-of-concept photonic-PUF based on a silicon photonic moiré QCI which was instantiated *N* = 3 times^15^. This replication allowed for *N* = 3 unique device authentications and *M* = *N*(*N − *1) = 6 inter-device comparisons or false authentication attempts. In this report, we further extend our research to a substantial *N* = 56 device instantiations realized in batches of 28 devices across two different fabrication facilities, with each instantiation and fab utilizing an exact copy of the same underlying QCI design. Semi-automated PUF measurements followed by digital key extraction enable *N* = 56 unique authentications and *M* = *N*(*N − *1) = 3080 inter-device comparisons or false authentication attempts to be performed. These photonic-PUF characterizations enable estimation of the authentication error rate (AER), false authentication rate (FAR), and probability of cloning (POC) as a function of the analysis parameters and/or authentication technique (e.g. Hamming vs. correlation based). Our results provide strong evidence of device uniqueness and unclonability and highlight disordered integrated photonics as a promising and scalable paradigm for realizing hardware security solutions.

## Approach

**Figure 1:**
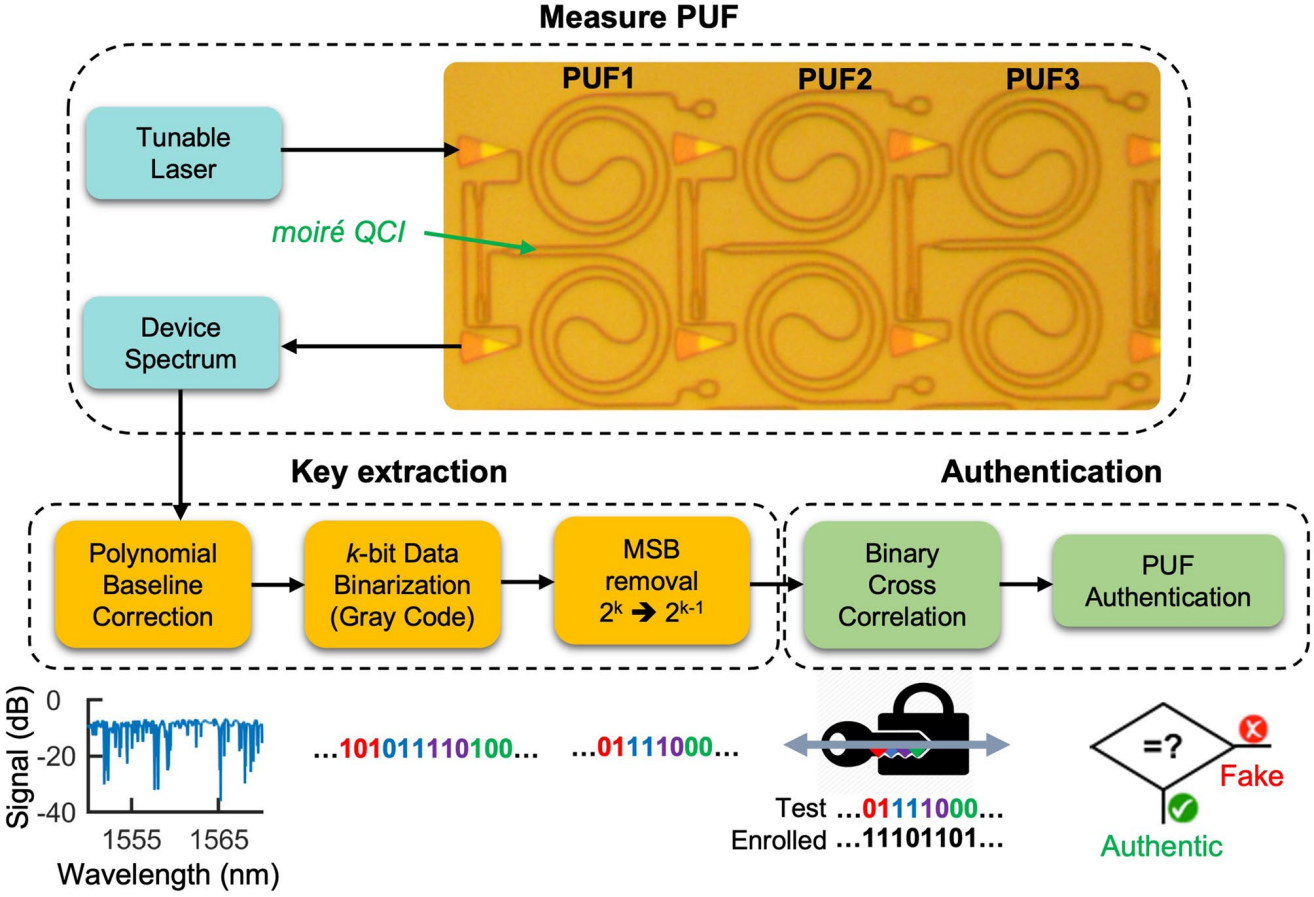
QCI PUF architecture and authentication framework: post-processing technique is shown involving conversion of spectral response to binary sequence, followed by binary cross correlation analysis to achieve proper authentication.

Fig. 1 illustrates our QCI based PUF and secure authentication framework. The QCI design is described in detail in Ref.^15^. Briefly, within each arm of the interferometer are waveguide spirals that contain identically designed silicon photonic quasicrystals which lack translational symmetry and support Aubry-André analyticity breaking^20^ and a 1D localization/delocalization transition^21^. The randomized nature of each photonic-PUF’s transmission spectrum is derived the QCI design being highly sensitized to distributed fabrication induced imperfections, such as nanoscale errors in waveguide width, which modulate the effective index profile of each quasicrystal and can induce transitions from delocalized waveguiding to localized resonant behavior. By designing our structures to include regions with narrow grating teeth and a small sidewall modulation depth of +/− 20 nm, we intentionally maximize the relative impact that nanoscale imperfections and natural spatially distributed fabrication disorder impart onto the realized devices^22^. Meanwhile, the grating couplers and single-mode waveguides act as polarization and mode filters which ensure the PUF response is insensitive to drifts or variations in probing conditions (e.g. polarization, angular or spatial alignment). In addition to enabling compact footprint and CMOS compatibility, the integrated nature of the device provides inherent robustness or reliability advantages over free-space or fiber based optical PUFs which may be highly sensitized to probing or environmental conditions^15^.

In this work, half, or *N*//2 = 28, of the identically designed PUF devices, were co-fabricated in two separate fabrication runs at (1) University of Washington Nanofabrication Facility and (2) Applied Nanotools fabrication facility, referred to here as ‘Fab 1’ and ‘Fab 2’ respectively (see Methods). As illustrated in Fig. 1, a digital key is extracted from each photonic-PUF through a series of steps. First, the device transmission spectra are collected with a tunable laser (Agilent 81600B) with a 10 pm resolution. The slowly varying spectral envelope associated with the grating couplers is then removed using a polynomial baseline correction. Note: spectra for all 56 devices are available in the supplementary information Fig. S1. The spectral features remaining after baseline correction are then purely associated with the photonic-PUF under test. The spectra are then binarized in gray code by rescaling the log scale transmission loss into a range between 0 and 2^ k^ − 1, where *k* is the number of bits for binarization. Due to limited entropy in the most significant bit (MSB) we remove the MSB which improves the approximate equiprobability of ‘0’s and ‘1’s in the extracted binary keys and brings the inter-chip fractional Hamming distance nearer to 0.5. The total bit length *L* of each photonic-PUF derived key is then *L* = *K*(*k − *1) where *K* is the number of wavelength samples. In our case, a 35 nm spectral wavelength window (1540–1575 nm) with a resolution of 10 pm results in *K* = 3500 and choosing *k* = 3 produces a key length of *L* = 7000 bits for each PUF.

## Results

**Figure 2:**
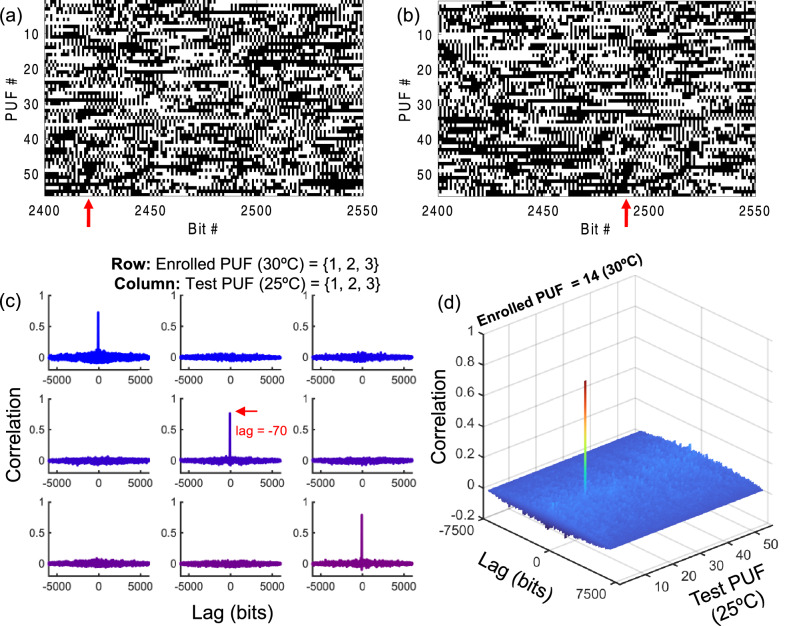
Digital keys and cross-correlation analysis. Visualization of a 150 bit subset of the binary keys generated from all 56 PUFs extracted from measurements at (**a**) 25 °C and (**b**) 30 °C; the red arrow indicates the 70 bit lag observed due to the spectral shift over temperature. (**c**) Cross-correlation analysis depicting the normalized correlation coefficient between the enrolled PUF at 30 °C and test PUF at 25 °C versus lag for selected PUFs 1–3. (**d**) Example cross-correlation analysis of enrolled PUF 14 versus all 56 test PUFs verifying the uniqueness and authenticity of the enrolled device.

To investigate device authentication all devices were remeasured approximately two days after their initial room temperature measurement at a secondary temperature (30 °C). A subset of the binary keys extracted for all 56 PUFs for *k* = 3 at 25 °C and 30 °C are visualized in Fig. 2a, b respectively. Due to silicon’s thermo-optic effect shifting the spectral response of each device in the wavelength domain over temperature, a corresponding shift in the bit sequence of each key is also observed. Experimentally we observe a shift of 70 bits (Fig. 2) indicating a thermo-optic wavelength shift of ~ 35 pm, which is in close agreement with prediction based on silicon’s thermo-optic coefficient of 1.86 × 10^−4^ RIU/K^23^ and a waveguide transverse confinement factor in silicon near ~ 0.88.

To evaluate authenticity or uniqueness between an enrolled key x(n) from the database and a new test key y(n), we measure the similarity between the two keys while simultaneously mitigating the influence of thermo-optic effects. Previously we have reported one analysis approach based on a “sliding key” Hamming distance (HD) computation, wherein the fractional HD is computed while shifting the test key relative to the enrolled key, with the output HD reported as the minimum fractional HD value obtained across all key lags^15^. A second and more standardized approach, evaluated here, would be to simply compute the normalized cross-correlation between the enrolled key, x(n), and the test key, y(n), and to record the maximum normalized cross-correlation value *C*_*xy*_ according to^24^:

$$C_{xy} = \max \left\{ {\frac{{R_{xy} \left( m \right)}}{{\sqrt {R_{xx} \left( 0 \right)R_{yy} \left( 0 \right)} }}} \right\}$$

where the unnormalized cross-correlation *R*_*xy*_(*m*) as a function of lag *m* is defined according to:

$$R_{xy} \left( m \right) = \left\{ {\begin{array}{*{20}l} {\sum\nolimits_{n = 1}^{L - m} x (n + m)y\left( n \right),\quad m \ge 0} \hfill \\ {\sum\nolimits_{n = 1}^{L + m} y (n - m)x\left( n \right),\quad m < 0} \hfill \\ \end{array} } \right.$$

Unlike a single HD or correlation computation, this cross-correlation based analysis naturally mitigates for any bit shifts that arise from the thermo-optic drift of the PUF’s spectral signature. This approach is expected to work effectively for large temperature drifts, e.g. +/− 30 °C^15^. In general however, the thermo-optic wavelength shift is proportional to the operating wavelength. Thus for optimal performance at extreme temperature drifts the cross-correlation approach could be modified by effectively scaling/stretching rather than simply shifting the wavelength axis in order to minimize intra-chip variation^15^. In addition to temperature drifts, it is plausible that device spectra from front-end to end-of-line or after packaging/assembly could exhibit subtle differences due to stress induced distortion. Although stress effects are not empirically examined in this work, based on existing literature^25,26^, we expect the stress-optic effect to be significantly smaller than the thermo-optic effect and could be similarly mitigated by the cross-correlation approach.

Fig. 2c, d illustrate the cross-correlation results for selected PUFs and confirm that distinct PUF keys are both uncorrelated and aperiodic. To facilitate arithmetic computation of the cross-correlation from a logical bit sequence, we assign logical ‘1’ to a positive variable *a* and logical ‘0’ to its negative, − *a*. For an ideally unbiased sequence with equiprobability of ‘0’ or ‘1’, this approach naturally removes the DC component of the signals. Note: a resulting correlation value *C*_*xy*_ near 1 or − 1 indicates strong correlation or anti-correlation respectively, while *C*_*xy*_ near 0 indicates signals that are uncorrelated. The aperiodic versus periodic nature of a given key is evaluated by identifying either only one spike or multiple spikes respectively from the cross-correlation or cross-autocorrelation.

**Figure 3:**
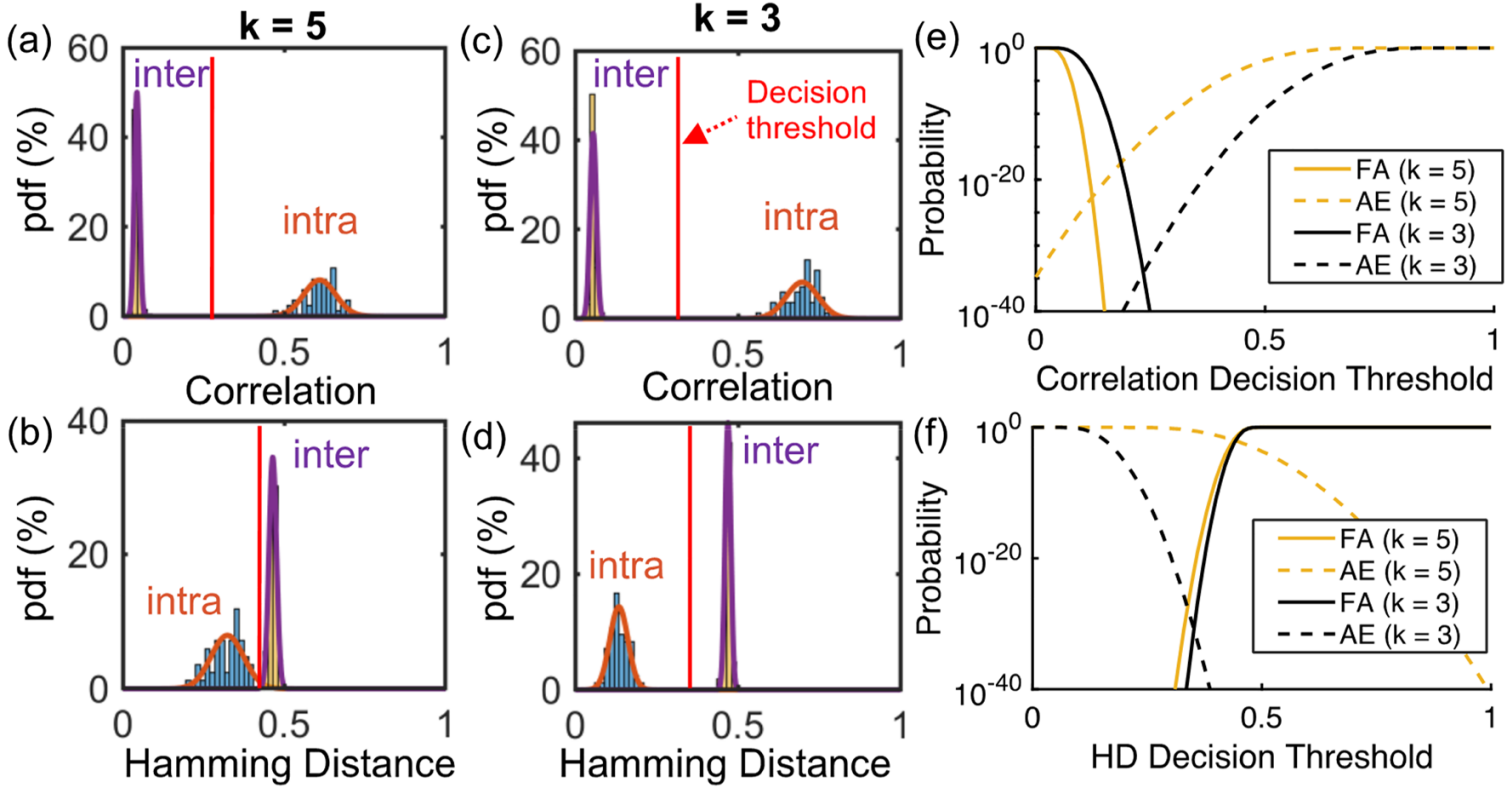
(**a**) Correlation-based authentication with k = 5, (**b**) HD based authentication with k = 5, (**c**) correlation-based authentication with k = 3, (**d**) HD based authentication with k = 3. Cumulative distribution functions indicating the probabilities of false authentication (FA) or authentication error (AE) as a function of decision threshold for (**e**) correlation based and (**f**) HD based authentication.

Next, we expand our analysis to all 56 PUFs and test for device authenticity by enrolling each key measured at 25 °C and comparing against all 56 test keys measured at 30 °C, allowing us to examine *N* = 56 ‘intra-chip’ authentication attempts and *N(N-1)* = 3080 ‘inter-chip’ false authentication attempts. To explore potential trade-offs between PUF key size and the reliability of each analysis technique (e.g. HD or correlation), we examined results for *k* values from 2 to 5 resulting in key sizes ranging from *L* = 1750 to 14,000 (supplementary Fig. S2). A summary of the correlation and HD based authentication results for *k* = 3 and 5 are reported in Fig. 3. As shown in Fig. 3a, b, the cross-correlation technique effectively distinguishes between fake and authentic devices for both key lengths as the inter-chip and intra-chip distributions are well isolated. For example, a correlation decision threshold near ~ 0.25 could be used to confidently distinguish between authentic versus fake devices with an experimentally observed false authentication rate (FAR) of 0% and authentication error rate (AER) of 0%. The HD technique also works effectively for *k* = 3, but exhibits a degradation in AER performance for *k* = 5 as indicated in Fig. 3c, d. These results suggest the HD method is more sensitive than the cross-correlation to bit errors which increase as the PUF spectra are digitized with higher resolution.

From the measured inter-chip and intra-chip probability density functions (pdfs), we then estimate the probabilities of false authentication (FA) and authentication error (AE) as a function of the decision threshold by computing the corresponding cumulative distribution functions (cdf) as reported in Fig. 3e, f. The probability of false authentication effectively provides an estimate of the PUF cloning probability. In the case where our PUF keys are authenticated using cross-correlation with *k* = 3 and 5, a decision threshold of 0.25 corresponds to estimated POC values below 10^−30^ and 10^−40^ respectively. The HD based analysis indicates a similar degree of unclonability, which suggests the primary benefits of the cross-correlation technique are its straightforward implementation, computational efficiency^24^, and improved intra-chip reliability, particularly for larger *k*.

**Figure 4:**
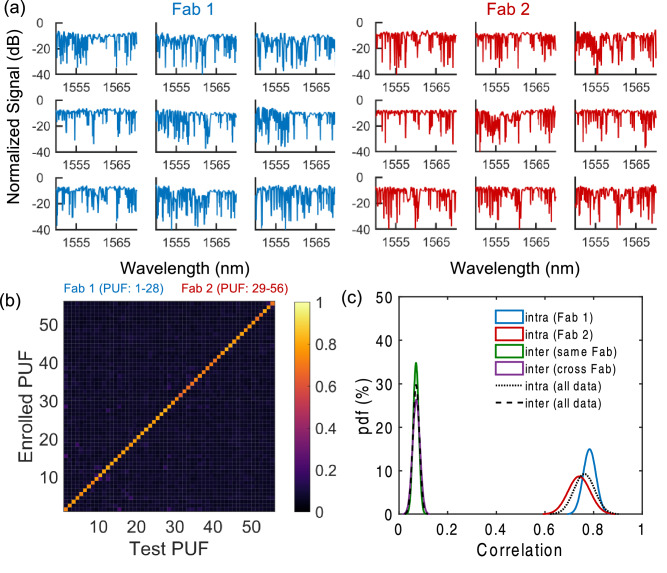
Cross-fab analysis: (**a**) QCI PUF spectra for a selected subset of 9 out of 28 PUFs from each Fab; (**b**) summary of cross-correlation based authentication results (k = 3) for all 56 PUFs; and (**c**) intra-chip and inter-chip distributions obtained when comparing PUF keys across or within each Fab.

Lastly, we summarize and breakdown our results according to the originating fabrication facility, with PUFs 1–28 corresponding to ‘Fab 1’ and PUFs 29–56 corresponding to ‘Fab 2’. As indicated by inspecting QCI PUF spectra from each fab (Fig. 4a and Supplementary Fig. S1), all QCIs provide randomized spectral features in the same working spectral window with similar extinction ratios. This indicates the processes are approximately matched in terms of propagation loss and the nominal waveguide dimensions which affect the nominal effective index and Bragg wavelengths of the constituent moiré sub-lattices used to construct the QCI. The results also qualitatively suggest a similar degree of nanoscale fabrication induced disorder is naturally present in each process. Despite these similarities, we found all 56 PUFs to be unique and uncorrelated to one-another as noted in results from Fig. 3 and summarized in Fig. 4b. Moreover, the uncorrelated nature of each distinct PUF is not found to exhibit any dependence on the fabrication facility, as the mean inter-chip correlation coefficient (maximum cross-correlation) is unchanged when comparing devices from the same fab (μ = 0.07) versus comparing devices across fabs (μ = 0.07) as shown in Fig. 4c. In other words, devices from both fabs were measured to be equally unclonable. The mean intra-chip correlation coefficient, however, does exhibit a small dependence on the fabrication facility, with devices originating from ‘Fab 1’ being authenticated with a higher mean correlation coefficient (μ = 0.78) than devices originating from ‘Fab 2’ (μ = 0.74). This however does not impact the empirically measured AER, which is observed to be 0% for devices from each fabrication facility.

## Conclusion

Our findings support the conclusion that QCI based silicon photonic-PUFs are a scalable solution for secure authentication in the untrusted supply chain. Compared to prior works typically comparing on the order of 10 inter-chip PUF signatures, this work compares > 10^3^ inter-chip PUF signatures. We observe zero authentication errors (out of N = 56 attempts) and zero false authentications (out of M = 3088 attempts). These results are achieved from devices replicated from the exact same PUF photonic circuit design across two different fabrication facilities. The size of this dataset allows us to empirically test the unclonability of our photonic-PUFs and to estimate the probability of cloning at less than 10^−30^. As such, this work provides an important step toward scalable implementation of photonic-PUFs in practical hardware authentication applications. These photonic-PUFs are also attractive for chip identification applications since they provide chip-unique signatures which could be used to identify and track parts from front-end wafer processing through to packaging and/or deployment within the untrusted supply chain, effectively serving as unforgeable and tamper-proof lot, wafer, and/or die identifiers. Furthermore, we anticipate related types of photonic-PUF structures, based on integrated photonics, can be scalably employed in active optoelectronic, all-optical, or quantum readout schemes to facilitate applications such as remote authentication and secure communication.

## Methods

*Device fabrication and testing* For this work, device fabrication and measurement was performed through the edX UBCx Phot1x Silicon Photonics Design, Fabrication and Data Analysis course (organized by L. Chrostowski)^27^. Half, or *N*/2 = 28, of the identically designed PUF devices were co-fabricated in two separate fabrication runs at the University of Washington nanofabrication facility (WNF or ‘Fab 1’) and Applied Nanotools (ANT or ‘Fab 2’) fabrication facility. *Applied Nanotools, Inc. NanoSOI process* The NanoSOI MPW fabrication process by Applied Nanotools Inc. (http://www.appliednt.com/nanosoi; Edmonton, Canada) is based on direct-write 100 keV electron beam lithography technology. Silicon-on-insulator wafers of 200 mm diameter, 220 nm device thickness and 2 µm buffer oxide thickness are used as the base material for the fabrication. After an initial wafer clean using piranha solution (3:1 H_2_SO_4_:H_2_O_2_) for 15 min and water/IPA rinse, hydrogen silsesquioxane (HSQ) resist was spin-coated onto the substrate and heated to evaporate the solvent. The photonic devices were patterned using a Raith EBPG 5000+ electron beam instrument using a raster step size of 5 nm. The exposure dosage of the design was corrected for proximity effects that result from the backscatter of electrons from exposure of nearby features. Shape writing order was optimized for efficient patterning and minimal beam drift. After the e-beam exposure and subsequent development with a tetramethylammonium sulfate (TMAH) solution, the devices were inspected optically for residues and/or defects. The chips were then mounted on a 4″ handle wafer and underwent an anisotropic ICP-RIE etch process using chlorine after qualification of the etch rate. The resist was removed from the surface of the devices using a 10:1 buffer oxide wet etch, and the devices were inspected using a scanning electron microscope (SEM) to verify patterning and etch quality. A 2.2 µm oxide cladding was deposited using a plasma-enhanced chemical vapour deposition (PECVD) process based on tetraethyl orthosilicate (TEOS) at 300ºC. Reflectometry measurements were performed throughout the process to verify the device layer, buffer oxide and cladding thicknesses before delivery. *Washington Nanofabrication Facility (WNF) silicon photonics process* The devices were fabricated using 100 keV Electron Beam Lithography^28^. The fabrication used silicon-on-insulator wafer with 220 nm thick silicon on 3 μm thick silicon dioxide. After a solvent rinse and hot-plate dehydration bake, hydrogen silsesquioxane resist (HSQ, Dow-Corning XP-1541-006) was spin-coated at 4000 rpm, then hotplate baked at 80 °C for 4 min. Electron beam lithography was performed using a JEOL JBX-6300FS system operated at 100 keV energy, 8 nA beam current, and 500 µm exposure field size. The machine grid used for shape placement was 1 nm, while the beam stepping grid, the spacing between dwell points during the shape writing, was 6 nm. An exposure dose of 2800 µC/cm2 was used. The resist was developed by immersion in 25% tetramethylammonium hydroxide for 4 min, followed by a flowing deionized water rinse for 60 s, an isopropanol rinse for 10 s, and then blown dry with nitrogen. The silicon was removed from unexposed areas using inductively coupled plasma etching in an Oxford Plasmalab System 100, with a chlorine gas flow of 20 sccm, pressure of 12 mT, ICP power of 800 W, bias power of 40 W, and a platen temperature of 20 °C, resulting in a bias voltage of 185 V. During etching, chips were mounted on a 100 mm silicon carrier wafer using perfluoropolyether vacuum oil. Cladding oxide was deposited using plasma enhanced chemical vapor deposition (PECVD) in an Oxford Plasmalab System 100 with a silane (SiH4) flow of 13.0 sccm, nitrous oxide (N2O) flow of 1000.0 sccm, high-purity nitrogen (N2) flow of 500.0 sccm, pressure at 1400mT, high-frequency RF power of 120 W, and a platen temperature of 350C. During deposition, chips rest directly on a silicon carrier wafer and are buffered by silicon pieces on all sides to aid uniformity. *Device measurement*: Semi-automated grating coupled device measurements were performed at The University of British Columbia. A tunable laser (Agilent 81600B) and optical power meter (Agilent 81635A) were used to capture device spectra over the range 1500–1600 nm in 10 pm steps.

## Supplementary Information

Supplementary Figures.

## Data Availability

The datasets generated during and/or analysed during the current study are available from the corresponding author on reasonable request.
