# Supplementary material for: Scalable and CMOS compatible silicon photonic physical unclonable functions for supply chain assurance
Source: Sci Rep. 2022 Sep 19;12:15653. doi: 10.1038/s41598-022-19796-z (PMC9485222; doi:10.1038/s41598-022-19796-z)
Supplement: Supplementary file 1 — Supplementary Figures. [file 41598_2022_19796_MOESM1_ESM.pdf]

Supplementary Information for: *Scientific Reports* manuscript "Scalable and CMOS compatible silicon photonic physical unclonable functions for supply chain assurance"

Farhan Bin Tarik, Azadeh Famili, Yingjie Lao and Judson D. Ryckman*
Holcombe Department of Electrical and Computer Engineering, Clemson University, Clemson, South Carolina, 29634, USA
*jryckma@clemson.edu

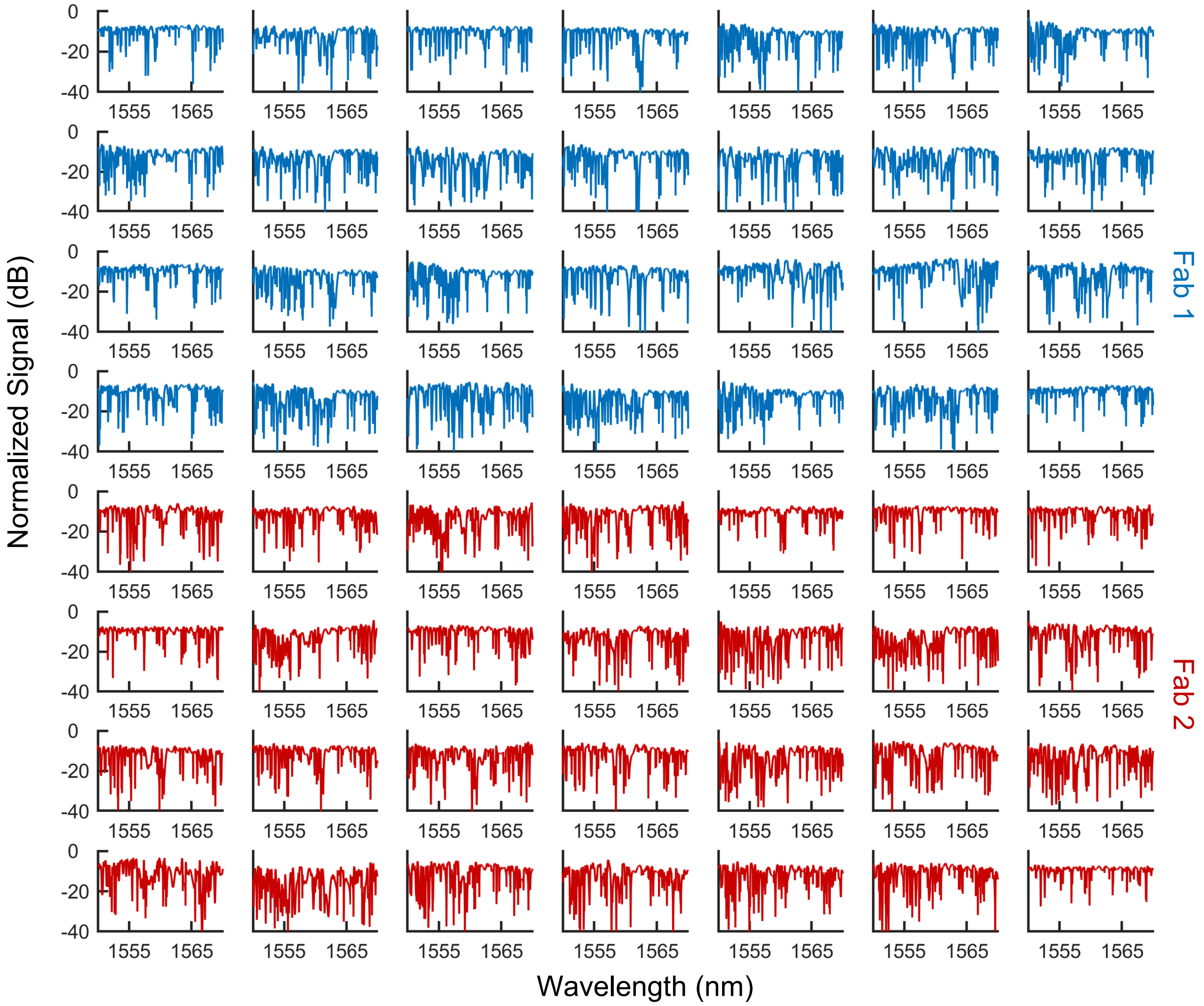

Fig. S1. Measured spectra shown after baseline correction for all 56 PUFs. (PUF1 in top left corner and PUF 56 in bottom right corner).

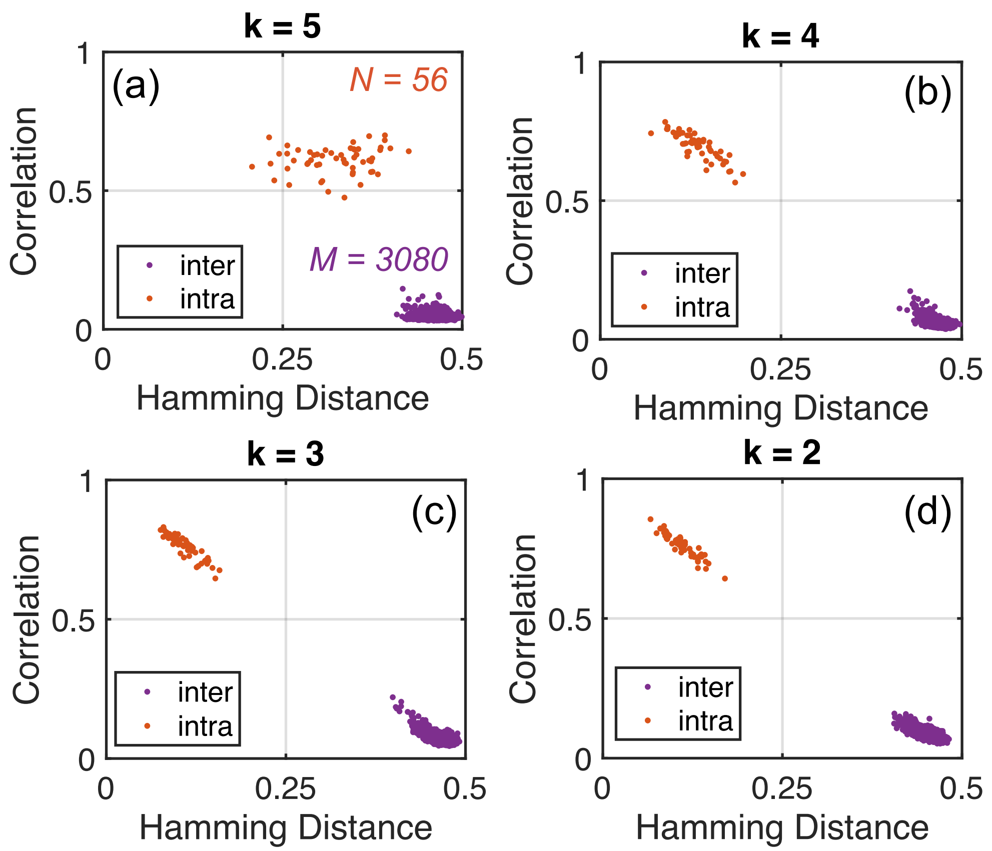

	Intra (Correlation)		Inter (Correlation)		Intra (Hamming)		Inter (Hamming)	
	μ	σ	μ	σ	μ	σ	μ	σ
**k = 5**	0.60968	0.049232	0.04372	0.00797	0.32364	0.05024	0.46438	0.0115
**k = 4**	0.69678	0.048932	0.05521	0.00962	0.13392	0.02782	0.46888	0.0086
**k = 3**	0.762	0.043006	0.07008	0.01340	0.1093	0.02087	0.46543	0.00977
**k = 2**	0.76113	0.043201	0.08804	0.01362	0.11035	0.02196	0.44944	0.0104

Fig S2. (Top) Correlation vs. HD results for (a) k = 5, (b) k = 4, (c) k = 3, and (d) k = 2. The correlation coefficient approaches 1 (or 0) as HD approaches 0 (or 0.5). A clear decision threshold cannot be drawn for HD method with k = 5, whereas one can be drawn using the correlation method. (Bottom) summary table of measured means and standard deviations.
